# Polyaniline-based 3D network structure promotes entrapment and detection of drug-resistant bacteria

**DOI:** 10.1186/s40580-023-00370-w

**Published:** 2023-05-27

**Authors:** Younseong Song, Nahyun Park, Da Ae Jo, Jueun Kim, Dongeun Yong, Jayeon Song, Yoo Min Park, Seok Jae Lee, Yong Tae Kim, Sung Gap Im, Bong Gill Choi, Taejoon Kang, Kyoung G. Lee

**Affiliations:** 1 Department of Chemical and Biomolecular Engineering, Korea Advanced Institute of Science and Technology, Daejeon, 34141 Republic of Korea; 2 Center for Nano Bio Development, National Nanofab Center (NNFC), Daejeon, 34141 Republic of Korea; 3 Department of Laboratory Medicine and Research Institute of Bacterial Resistance, Yonsei University College of Medicine, Seoul, 03722 Republic of Korea; 4 Center for Systems Biology, Massachusetts General Hospital, Boston, MA 02114 USA; 5 Department of Radiology, Massachusetts General Hospital, Harvard Medical School, Boston, MA 02114 USA; 6 Bionanotechnology Research Center, Korea Research Institute of Bioscience and Biotechnology (KRIBB), Daejeon, 34141 Republic of Korea; 7 Department of Chemical Engineering & Biotechnology, Tech University of Korea, Siheung-Si, 15073 Republic of Korea; 8 Department of Chemical Engineering, Kangwon National University, Samcheok, 25913 Republic of Korea; 9 School of Pharmacy, Sungkyunkwan University (SKKU), Suwon-Si, 16419 Republic of Korea

**Keywords:** Nanotopology, Nanopillar, Drug-resistant bacteria, On-site diagnostics, Polyaniline

## Abstract

Sensitive and accurate capture, enrichment, and identification of drug-resistant bacteria on human skin are important for early-stage diagnosis and treatment of patients. Herein, we constructed a three-dimensional hierarchically structured polyaniline nanoweb (3D HPN) to capture, enrich, and detect drug-resistant bacteria on-site by rubbing infected skins. These unique hierarchical nanostructures enhance bacteria capture efficiency and help severely deform the surface of the bacteria entrapped on them. Therefore, 3D HPN significantly contributes to the effective and reliable recovery of drug-resistant bacteria from the infected skin and the prevention of potential secondary infection. The recovered bacteria were successfully identified by subsequent real-time polymerase chain reaction (PCR) analysis after the lysis process. The molecular analysis results based on a real-time PCR exhibit excellent sensitivity to detecting target bacteria of concentrations ranging from 10^2^ to 10^7^ CFU/mL without any fluorescent signal interruption. To confirm the field applicability of 3D HPN, it was tested with a drug-resistant model consisting of micropig skin similar to human skin and *Klebsiella pneumoniae* carbapenemase-producing carbapenem-resistant *Enterobacteriaceae* (KPC-CRE). The results show that the detection sensitivity of this assay is 10^2^ CFU/mL. Therefore, 3D HPN can be extended to on-site pathogen detection systems, along with rapid molecular diagnostics through a simple method, to recover KPC-CRE from the skin.

**Graphical Abstract:**

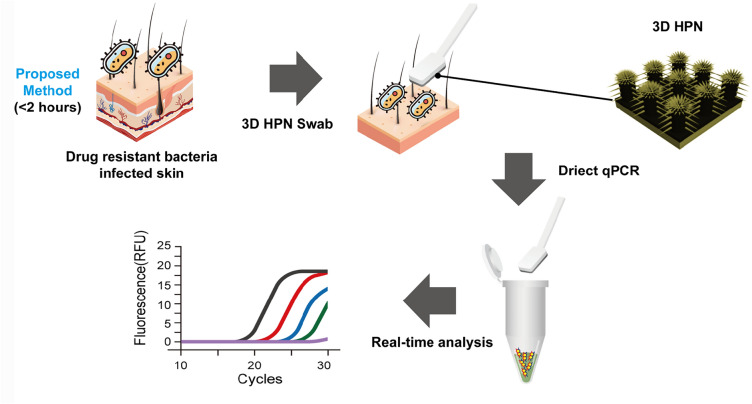

## Introduction

Since the emergence of antibiotics, several types of multi-drug-resistant (MDR) bacteria have appeared and become a major public health concern [1–3]. Recently, this has become a major issue because several COVID-19 patients in hospitals have been exposed to MDR bacteria since the outbreak of the COVID-19 pandemic [4–6]. MDR bacteria (i.e., also known as superbugs) cause approximately 700,000 deaths annually worldwide [5]. MDR bacteria are easily spread from infected patients via contaminated hands, skin, tools, and equipment in a hospital [7–11]. Therefore, to overcome such challenges, a sensitive, robust, and on-site diagnostic approach is highly required to realize the rapid identification of MDR bacteria and provide proper treatment guidelines to both doctors and patients [12–14].

To date, MDR bacteria are openly identified by culturing after antibiotic exposure [15, 16]. This method involves a sample swab for the recovery of bacteria from infected skin, culture, enrichment, and antibiotic susceptibility testing, which takes 2–5 days and requires highly trained personnel because of the complicated procedures [17]. More recently, molecular diagnosis has been considered one of the most accurate and reliable methods for detecting specific genes through nucleic acid amplification (i.e., the polymerase chain reaction, PCR) that directly detects antibiotic-resistant genes [18–21]. Although the PCR exhibits high analytical sensitivity, accuracy, and selectivity, it requires a highly effective capturing tool to recover target bacteria from infected skin and needs to have antibacterial characteristics to avoid potential secondary infection from the bacteria [22, 23].

Recently, nano-topological technologies have drawn significant attention for recovering and diagnosing pathogenic bacteria owing to their high surface area, controllable surface characteristics, mechanical, chemical, and thermal stability, and compatibility in molecular analysis [24–26]. In addition, various nanostructures and chemical modifications have been employed to entrap different types of pathogens and recover their nucleic acids via physicochemical interactions [27–31]. In particular, two-dimensional (2D) nanoarrays and three-dimensional (3D) hierarchical nanostructures exhibit excellent performance by introducing topographical interactions with extracellular matrices of bacteria [32–34]. We hypothesized that the synergetic convergence of the nano-topological capturing tool and molecular analysis technique allows for a simple, accurate, sensitive, and reliable method for detecting MDR bacteria.

Herein, we report an advanced technique for fabricating a 3D hierarchically structured polyaniline nanoweb (3D HPN) as an efficient MDR pathogenic bacteria-capturing tool with robust mechanical reliability for rubbing infected skin. The 3D nanonetworks consist of a polyurethane-based nanopillar (PUN) array and secondary growth of polyaniline nanofibers on nanopillars. The interfacial interactions between the extracellular organelles of MDR bacteria and the nanosurface of 3D HPN were investigated, and bacterial extracellular organelles were intensively investigated through scanning electron microscopy (SEM) and Fourier transform infrared spectroscopy (FTIR). To demonstrate the capturability of 3D HNP, *Klebsiella pneumoniae* carbapenemase-producing *carbapenem-resistant Enterobacteriaceae* (KPC-CRE) were chosen as the target MDR bacteria because it is one of the major concerns of public health due to its increasing prevalence, rapid regional dissemination, and high mortality rate ($40\pm 10\mathrm{\%}$) [35, 36]. Micropig skin was selected as a realistic model of human skin [36, 37]. In addition, the real-time PCR-based molecular analysis of 3D HNP successfully confirmed single bacterial cell capturability and mechanical reliability against shear stress. We expect that this new approach can be applied to the on-site diagnosis of MDR bacteria in the early stages of bacterial infection.

## Materials and methods

### Fabrication of 3D HPN

PUN array films were prepared by soft lithography using a silicon template during the UV-curing process [38, 39]. In addition, 20 nm thick titanium (Ti) and 200 nm thick gold (Au) were deposited on the surface of PUN films. 3D HPN films were prepared by synthesizing polyaniline on a nanopillar array of the PUN films, where polyaniline was grown using the dilute polymerization method. The PUN films were immersed in deionized (DI) water of 1-M perchloric acid (70%, Sigma-Aldrich), 0.1-M aniline (99.5%, Sigma-Aldrich), and 6-mM ammonium persulfate (98%, Sigma-Aldrich) molar concentrations. The polymerization was performed at 4 °C and incubated for 8 h. The 3D HPN films were gently washed with DI water and 70% ethanol several times.

### Characterization

The morphology and chemical state of the 3D HPN films were characterized by field-emission SEM (Hitachi S-4800, Japan). To investigate the interfacial interactions between 3D HPN and *E. coli* O157:H7, the cells were dropped onto the surface of 3D HPN, and then time-course FTIR spectra were collected using the JASCO FTIR-4600 in the range of 300‒4000 cm^‒1^ with the attenuated total reflectance technique.

### Entrapment and anti-release efficiency

The entrapment efficiency of 3D HPN was evaluated by inoculating serial dilutions of KPC-CRE solution from 10^4^ to 100 CFU/mL into the 3D HPN sheet (2 $\times$ 4 mm^2^). After 1-h incubation at 25 °C, a supernatant was carefully recovered from the 3D HPN sheet. The colony assay for the recovery solution was conducted to count colonies of bacteria remaining after entrapment. In detail, 100 μL of the recovery solution was spread to the solid agar plate. After incubation at 34 °C for 15 h, the number of colonies was counted in the solid agar plate. The number of entrapped bacteria was determined following the following equation:

$${\text{Entrapment}}\,{\text{efficiency}}\,\left( \% \right)\, = \,\left( {1 - \left( {{\text{N}}_{{\text{R}}} \,/\,{\text{N}}_{{{\text{NC}}}} } \right)} \right)\, \times \,100$$

where N_R_ denotes the number of colonies for the recovery solution after entrapment. N_NC_ denotes the number of colonies for the initial bacteria solution.

The anti-release efficiency of 3D HPN entrapping KPC-CRE was evaluated by rubbing this 3D HPN sheet onto a solid agar plate. Then, a colony assay was conducted by incubating the rubbed solid agar plate at 34 °C for 15 h. After incubation, the colonies were counted and used to calculate the anti-release efficiency below:

$${\text{Anti - release}}\,{\text{efficiency}}\,\left( \% \right)\, = \,\left( {1 - \left( {{\text{N}}_{{\text{B}}} /\left( {{\text{N}}_{{{\text{NC}}}} \, - \,{\text{N}}_{{\text{R}}} } \right)} \right)} \right)\, \times \,100$$

where N_B_ represents the number of colonies released from the 3D HPN sheet while rubbing it on the agar plate.

### Live/dead assay

The live/dead assay was tested by inoculating serial dilutions of KPC-CRE from 10^8^ to 10^0^ CFU/mL into the 3D HPN sheet (2 $\times$ 1 cm^2^). After 1-h incubation at 25 °C, a supernatant was carefully removed from the 3D HPN sheet, and the sheet was washed with DI water. KPC-CRE captured on the sheet was stained with SYTO 9 (Merck), and the sheet was washed with DI water. The stained bacteria were then imaged via laser scanning confocal microscopy after 15 min.

### PCR analysis of entrapped bacterial pathogens

KPC-CRE was provided by Severance hospital and was cultured in Luria–Bertani broth for 18 h at 37 ℃ in a shaking incubator. Strains were collected by rubbing 3D HPN once or twice on KPC-CRE colonies in an agar plate. KPC-CRE were lysed using a QuickExtract^™^ DNA Extraction Solution (epicenter) for 15 min at 98 ℃. For the molecular diagnosis of KPC-CRE, a Super bacteria KPC-CRE detection kit (Bio-TNS, Daejeon, Korea) was used. The PCR cocktail was prepared with a total 20 μL volume, including 10 μL of 2 × qPCR Premix (Bio-TNS), 6 μL of KPC-CRE kit, 2 μL of lysed KPC-CRE, and 2 μL of diethylpyrocarbonate water (Enzynomics). PCR amplification was performed using a T100 Thermal Cycler (Bio-Rad) under the conditions of initial denaturation at 95 ℃ for 10 min, 35 thermal cycles of 95 ℃ for 10 s, 60 ℃ for 10 s as specified in the Super bacteria KPC-CRE detection kit protocol provided by the manufacturer (Bio-TNS, Daejeon, Korea).

### Drug-resistant animal skin model

As the drug-resistant bacteria-infected animal skin, micropig Franz cell membrane (APURES, Republic of Korea)) was used in this study. KPC-CRE was selected as the target drug-resistant bacteria. Serial dilutions of KPC-CRE solution were prepared with concentrations of 10^2^, 10^4^, and 10^7^ CFU/mL. The prepared bacteria solution was spread into the micropig skin (3 $\times$ 3 cm^2^) to prepare the infected skin. After 1-h incubation at 25 °C, a supernatant was recovered from the 3D HPN sheet, followed by lysis using the QuickExtract^™^ DNA Extraction Solution for 15 min at 98℃. The lysates were immediately used in the real-time PCR assay to identify KPC-CRE.

## Results and discussion

### Synthesis of 3D HPN and its characterization

**Scheme 1:**
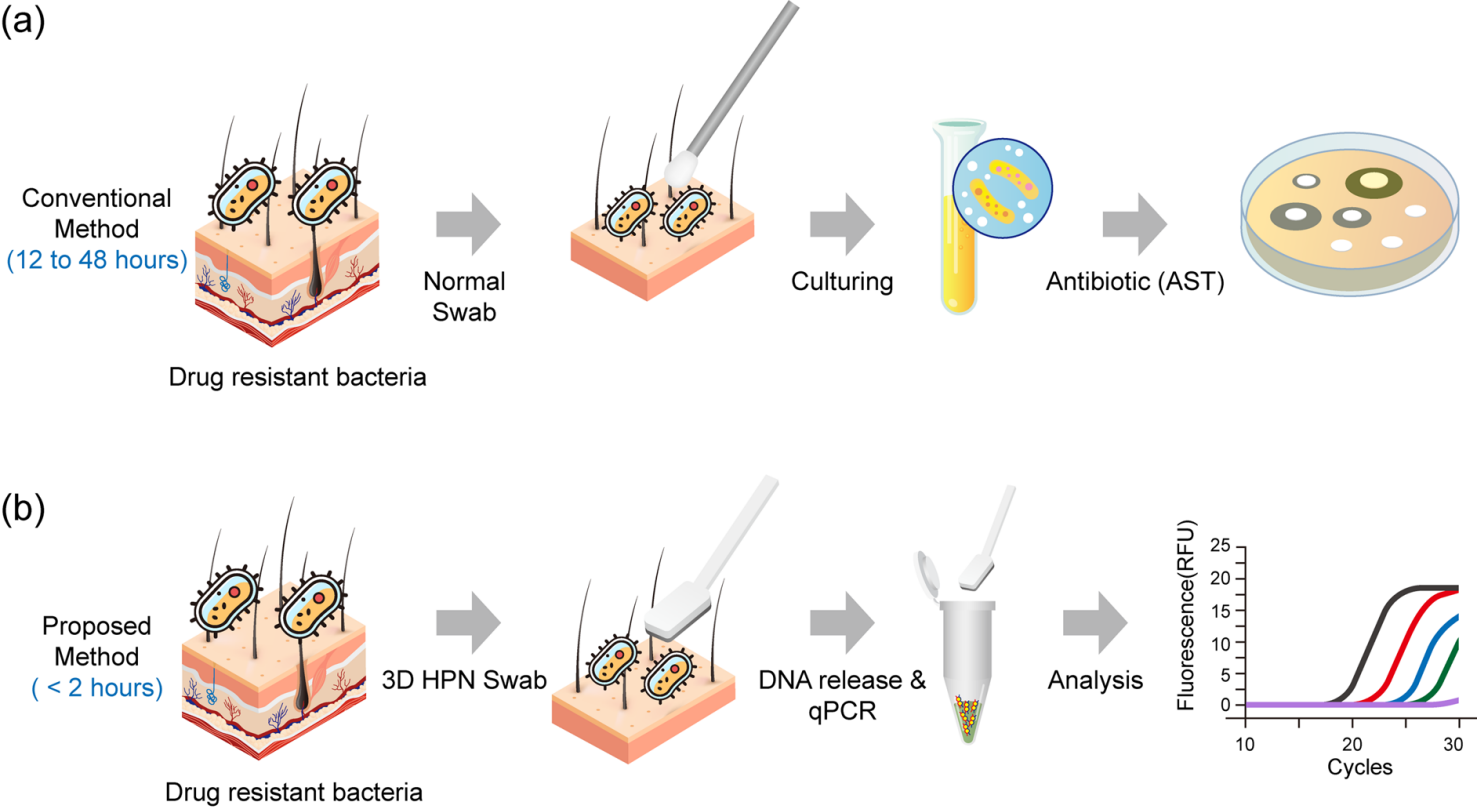
Schematic illustration of the overall process of **a** conventional and **b** proposed methods

The overall process of the conventional and proposed methods is depicted in Scheme 1a and b. The conventional method takes 12–48 h after the recovery of drug-resistant bacteria and culturing (Scheme 1a). However, the proposed method using 3D HPN is performed in less than 2 h, including the capture of drug-resistant bacteria and genetic analysis (Scheme 1b).

**Fig. 1:**
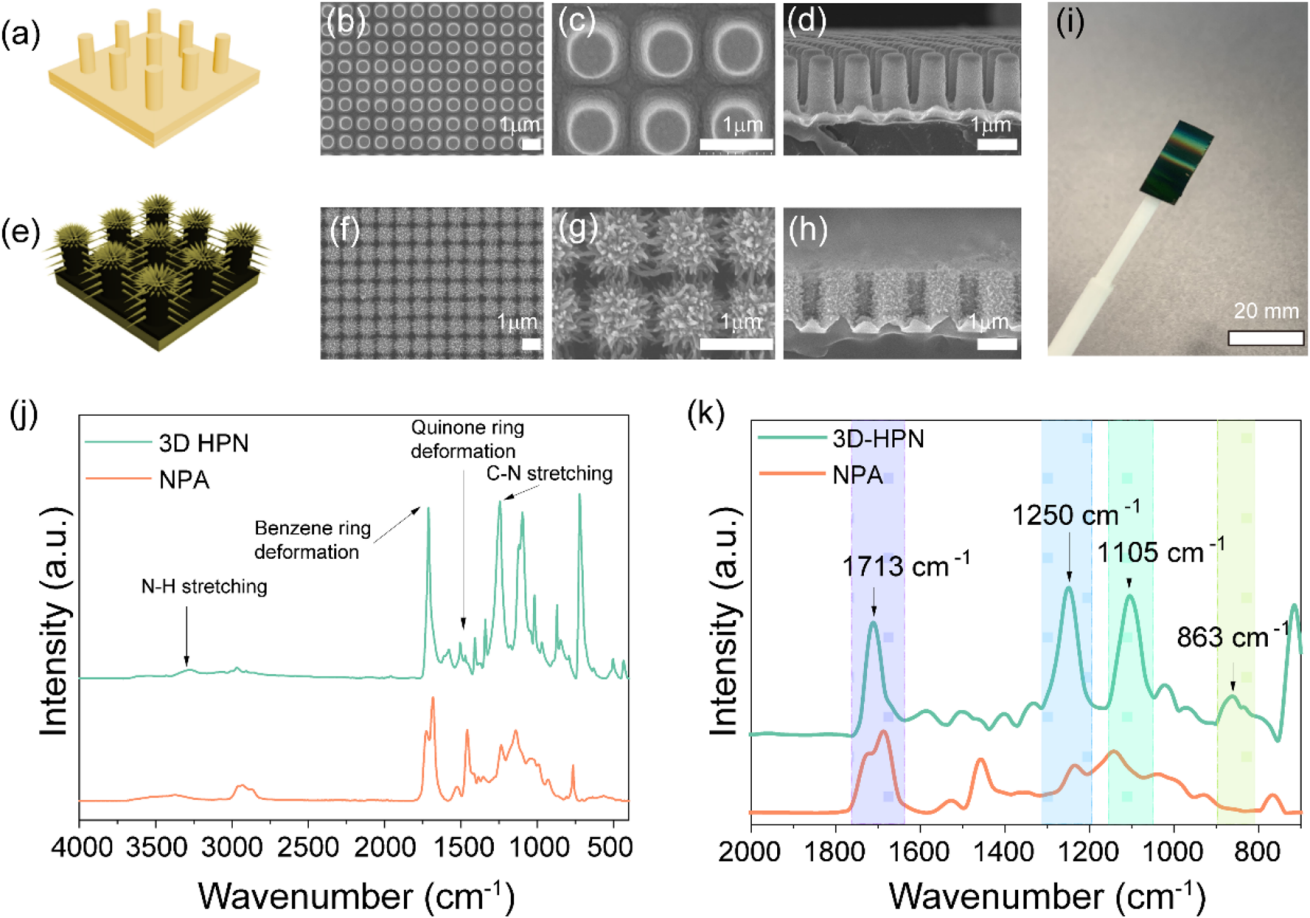
**a** Scheme of NPA and **b**–**d** SEM images of the top and cross-section of NPA. **e** Scheme of 3D HPN and **f**–**h** SEM images of the top and cross-section of 3D HPN. **i** Photograph of 3D HPN. **j** FTIR spectrum of 3D HPN and NPA. **k** Enlarged FTIR spectrum with characteristic peaks of polyaniline from 2000 to 750 cm^−1^

The 3D HPN film is fabricated through the replication of a nanopillar array (NPA) and the growth of polyaniline nanofibers on the NPA. The NPA is simply replicated from a nanohole-arrayed silicon wafer prepared through the photolithography and etching of the wafer using the blend of polyurethane acrylate and NOA63 and a polyethylene terephthalate (PET) film. The NPA has a diameter, height, and center-to-center distance of 500 nm, 1.25 μm, and 1 μm, respectively. Once the NPA is ready, 10-nm-thick titanium and 100-nm-thick gold layers are further coated on the NPA using the thermal evaporation method to grow a polyaniline nanofiber network. In addition, this dilute polymerization method allows us to fully cover the NPA and uniformly synthesize the polyaniline network between the NPA. Nano-scaled polyaniline networks are grown both on the top and sidewalls of the NPA, resulting in the successful construction of a 3D hierarchical nanonetwork around the NPA. SEM images indicated the successful fabrication of a uniform and highly ordered NPA and the further formation of a polyaniline-based spike-shaped nanonetwork around the NPA, as shown in Fig. 1a–d and e–h. Once the film is ready, it is transferred onto a handheld capturing tool to recover drug-resistant bacteria from the target surface (Fig. 1i). Similarly, polyaniline was characterized by a broad band near 3300 cm^−1^, indicating N–H stretching vibrations of aromatic amine, and bands near 1700 and 1500 cm^−1^ correspond to benzene and quinone ring deformations. The absorption peak near 1250 cm^−1^ is attributed to the stretching vibration of the C–N group of secondary aromatic amine. Peaks near 1100 and 800 cm^−1^ indicate the C–H in-plane bending and C-H aromatic out-of-plane bending modes, respectively (Fig. 1j and 1k).

### Entrapment and analysis of bacteria on 3D HPN

**Fig. 2:**
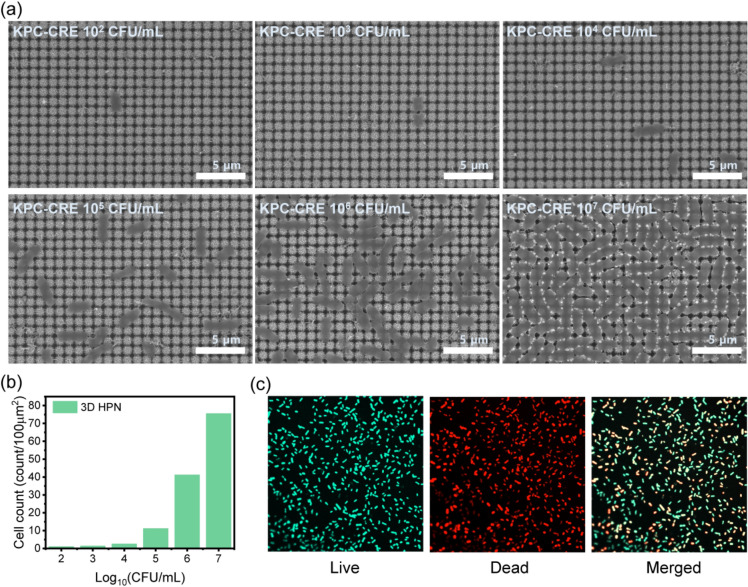
**a** SEM images of 3D HPN-entrapped KPC-CRE at a cell number of 10^2^–10^7^ CFU/mL. **b** The number of cells entrapped on 3D HPN. **c** Live/dead images of 10^5^ CFU/mL KPC-CRE entrapped in 3D HPN

For a better understanding of the capture performance of 3D HPN, the commonly known MDR bacteria of KPC-CRE were selected and exposed to the surface of 3D HPN. Different amounts of bacteria from 10^2^ to 10^7^ CFU/mL were prepared and dropped on the 3D HPN, respectively. After rinsing the surface with DI water, the morphological changes of 3D HPN were investigated via SEM, as shown in Fig. 2a. By observing the SEM images, the as-developed 3D HPN successfully captured the target bacteria with bacterial concentrations ranging from 10^2^ to 10^7^ CFU/mL. Furthermore, as the concentration of bacteria increased, the captured bacteria on 3D HPN also increased proportionally and were incorporated into the nanonetwork. More interestingly, most of the 3D HPN surface is fully covered with bacteria of bacterial concentrations of more than 10^7^ CFU/mL. Entrapment coverage displays a maximum of 75 counts per 100 μm^2^ when 10^7^ cells are loaded (Fig. 2b). In addition, a live/dead assay was performed to confirm the state of the bacteria entrapped on 3D HPN. Figure 2c shows several dead bacteria on 3D HPN after 1 h entrapment. This can help prevent secondary infections caused by contact with 3D HPN. Therefore, the as-developed 3D HPN is highly suitable for the effective capture of MDR bacteria for potential diagnostic analysis.

### Entrapment and anti-release performance evaluation of 3D HPN

**Fig. 3:**
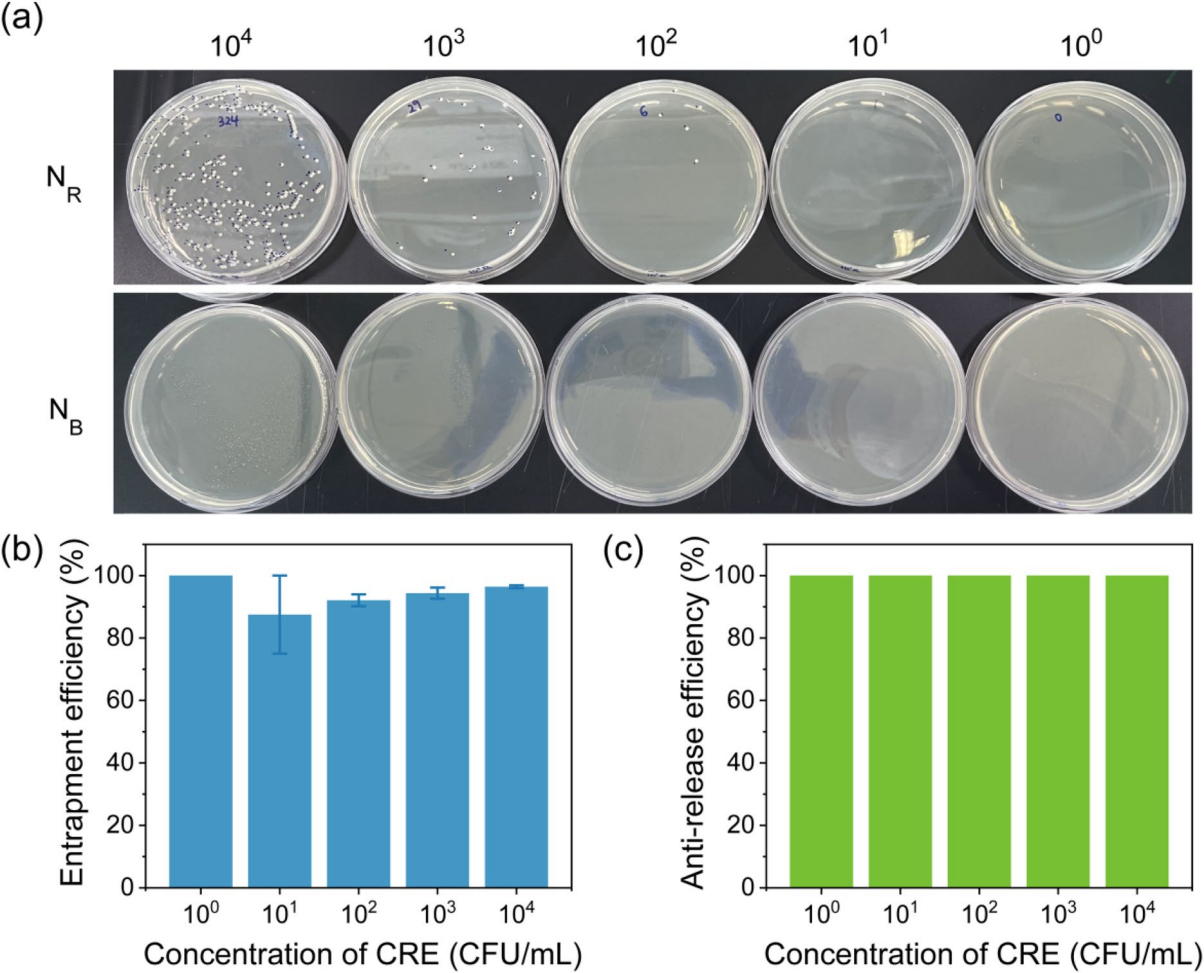
Entrapment and anti-release performance of 3D HPN against serial dilutions of KPC-CRE solutions. **a** Colony assay for the number of colonies remaining after 3D HPN entrapment (N_R_, above) and for the number of colonies released from 3D HPN (N_B_, below) depending on KPC-CRE concentrations ranging from 10^4^ to 10^0^ CFU/mL. **b** The entrapment efficiency of 3D HPN with KPC-CRE concentrations ranging from 10^0^ to 10^4^ CFU/mL. Entrapment efficiency (%) = (1-(N_R_/N_NC_))$\times$ 100. **c** Anti-release efficiency of 3D HPN with KPC-CRE concentrations ranging from 10^0^ to 10^4^ CFU/mL. Anti-release efficiency (%) = (1-(N_B_/(N_NC-_N_R)_))$\times$ 100. All experiments are conducted in triplicate

The entrapment performance of 3D HPN was evaluated using a colony assay. Serial dilutions of KPC-CRE solution from 10^4^ to 10^0^ were loaded onto the 3D HPN surface. After 1-h incubation, the remaining KPC-CRE was recovered, and a colony assay was performed. Figure 3a shows 324, 29, 1, and 0 colonies, which indicate the remaining KPC-CRE amount (N_R_) in the recovery solution after 3D HPN-entrapped the KPC-CRE solution with initial loading amounts of 10^4^, 10^3^, 10^2^, 10^1^, and 10^0^ CFU/mL. 3D HPN exhibits approximately 85% entrapment efficiency, which is determined in terms of N_R_ for KPC-CRE concentrations ranging from 10^4^ to 10^0^ CFU/mL (Fig. 3b).

To prevent secondary infection by KPC-CRE, it is crucial that 3D HPNs do not release bacteria that are trapped in their structures. Thus, the anti-release capability of 3D HPN plays a significant role in trapping and detecting KPC-CRE. We tested the anti-release performance of 3D HPN by rubbing KPC-CRE-entrapped 3D HPN sheets onto solid agar plates. The KPC-CRE-entrapped 3D HPN sheets are prepared through an entrapment experiment with different KPC-CRE concentrations ranging from 10^4^ to 10^0^ CFU/mL. The rubbed agar plates were incubated at 34 ℃ for 15 h to count the colonies (N_B_) of released bacteria from KPC-CRE-entrapped b-SNAP. As shown in Fig. 3a (below), the colony is not observed in any other KPC-CRE concentration. Therefore, 3D HPN exhibits 100% anti-release efficiency for the given KPC-CRE concentrations (Fig. 3c). This result shows that 3D HPN has a high anti-release capability and is applicable to KPC-CRE entrapment and detection systems.

### Practical applicability of 3D HPN for pathogenic bacterial entrapment and detection

**Fig. 4:**
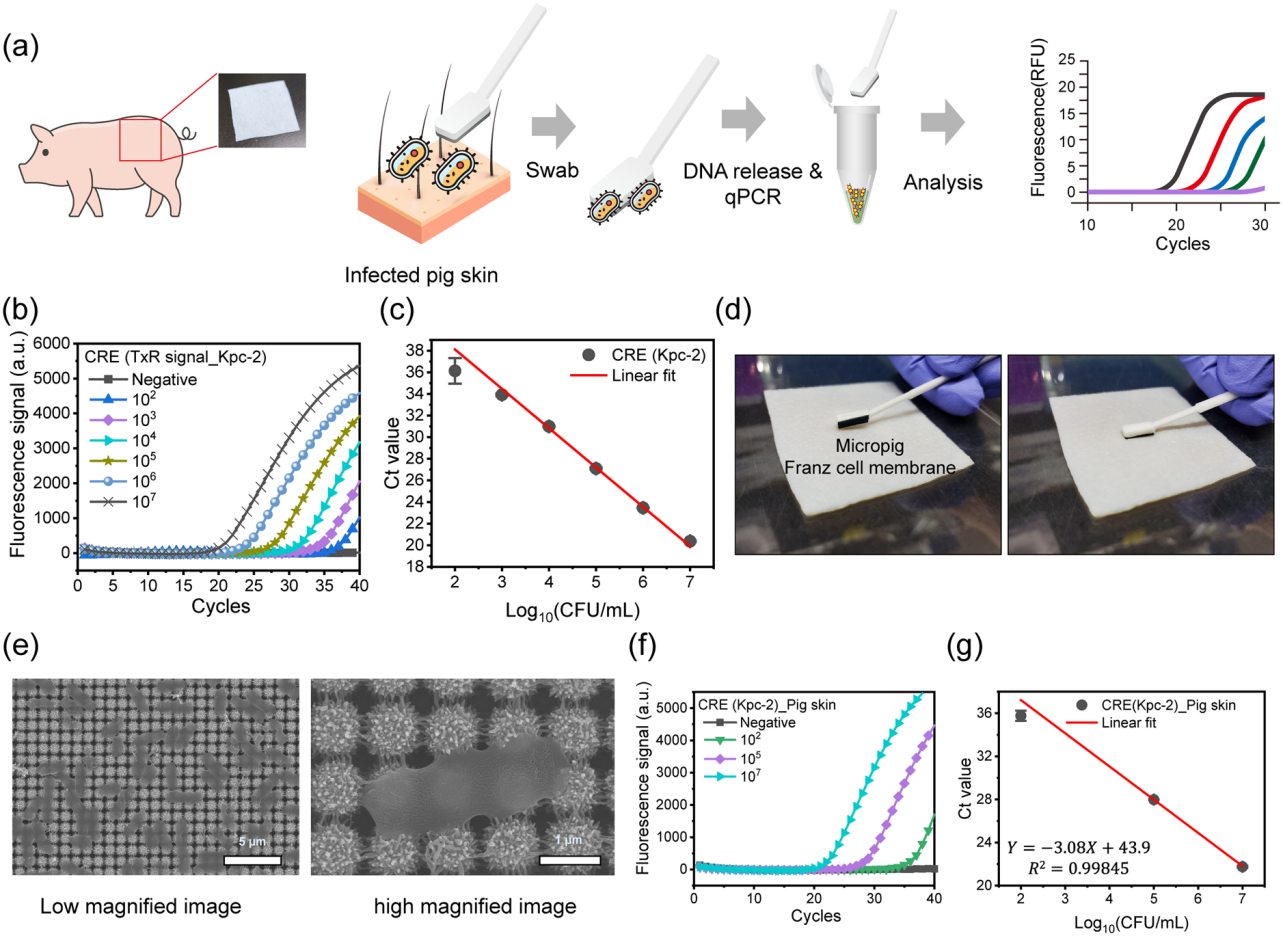
**a** Schematic workflow of KPC-CRE detection on infected micropig skin using 3D HPN tool-based real-time PCR. **b** Real-time PCR results and **c** the corresponding Ct values targeting the Kpc-2 gene of KPC-CRE entrapped in 3D HPN by loading KPC-CRE solutions at various concentrations ranging from 10^2^ to 10^7^ CFU/mL). **d** Digital images of KPC-CRE-infected micropig Franz cell membrane (i.e., micropig skin), 3D HPN tool (left), and the micropig skin being rubbed by 3D HPN (right). **e** SEM images of KPC-CRE captured by 3D HPN from micropig skin (left), and the magnified image (right). **f** Real-time PCR result and **g** corresponding Ct values of KPC-CRE captured by 3D HPN from the infected micropig skin at different KPC-CRE concentrations of 10^2^, 10^5^, and 10^7^ CFU/mL

To demonstrate the detection of MDR bacteria through 3D HPN tool-based real-time PCR, micropig skin is used as a realistic model (Fig. 4a). Micropig skin has characteristics similar to human skin, and it is typically used as a human skin model [40]. The KPC-CRE solutions are spread over the micropig skin, and the skin is rubbed by the 3D HPN tool, followed by a DNA-release process and real-time PCR analysis. Before the application to the micropig skin, the sensitivity of 3D HPN tool-based real-time PCR was tested (Fig. 4b). Serial dilutions of KPC-CRE solutions are deposited on 3D HPN sheets (4 $\times$ 10 mm^2^), and the KPC-CRE-entrapped 3D HPNs are immersed in the lysis buffer to release the DNA. The released DNA was quantitatively analyzed by a real-time PCR. Figure 4b shows the real-time PCR amplification signals, which exponentially increase with the number of cycles and regularly shift to the right with a decrease in initial injected KPC-CRE concentrations. The negative sample (i.e., 3D HPN without KPC-CRE) has no amplification curve, indicating that 3D HPN is compatible with real-time PCR processes, and this detection system is free from non-specific signals. The cycle threshold (Ct) values obtained from the real-time PCR plot show high sensitivity (LOD = 10^2^ CFU/mL) and linear correlation curves (${R}^{2}=0.9982$) with the concentrations (Fig. 4c).

The 3D HPN tool-based real-time PCR assay is applied to the micropig skin artificially infected by spiking KPC-CRE of 10^2^, 10^5^, and 10^7^ CFU/mL. The 3D HPN tool gently touched the micropig skin and rubbed the different amounts of KPC-CRE of 10^2^, 10^5^, and 10^7^ CFU/mL from the spiked micropig skin surface several times (Fig. 4d). After the recovery of 3D HPN from the tool, several KPC-CRE concentrations can be observed on the surface of 3D HPN, as shown in the SEM images (Fig. 4e). In addition, no significant changes in the 3D HPN morphology are observed before and after rubbing the skin. The KPC-CRE entrapped on the 3D HPN is lysed with a lysis buffer, and the lysates are amplified using a real-time PCR. Figure 4f shows the amplification plot of the different amounts of KPC-CRE spiked in the micropig skin. The amplification curves of each sample are right-shifted with decreasing initial KPC-CRE concentrations. The corresponding C_t_ values of KPC-CRE are plotted in Fig. 4g. The dynamic range of KPC-CRE was 10^2^–10^7^ CFU/mL. The regression curve shows a slope of -3.08 with a correlation coefficient (R^2^) of 0.99845, confirming the highly linear relationship between the C_t_ value and the logarithm of the initial KPC-CRE concentration spread on the micropig skin. Based on the slope of the regression curve, we derived the PCR efficiency of 111%, which is an approximately ideal value ranging between 90 to 110% [41]. Thus, we found that 3D HPN does not affect the efficiency of real-time PCR, thus demonstrating its compatibility with real-time PCR. The detection sensitivity of this assay is 10^2^ CFU/mL. Thus, our findings indicate the excellent directive KPC-CRE capturability and detectability performance of 3D HPN through skin rubbing. Interestingly, the strong adhesion force between KPC-CRE and 3D HPN provides a relatively convenient method for recovering KPC-CRE from the skin and easily recovering target genes for further analysis.

## Conclusions

In summary, we successfully fabricated a 3D HPN film for capturing MDR KPC-CRE from the skin surface. The 3D HPN films exhibited reliable mechanical and chemical characteristics for the effective capture of target bacteria by rubbing the skin. Furthermore, their unique 3D hierarchical structures allow for secure binding sites to avoid potential secondary infection by providing prominent antibacterial properties. The real-time PCR results show the effective capturability of 3D HPN over a broad range of cell concentrations. Furthermore, the direct bacteria capture from pig skin by 3D HPN confirmed that this tool can recover KPC-CRE bacteria from the skin and detect the bacteria for further gene-based diagnosis. Based on the obtained results, 3D HPN can be used for the capture of infectious bacteria and as an on-site detection system.

## Data Availability

The datasets used and/or analyzed during the current study are available from the corresponding author upon reasonable request.
